# Association between COVID-19 pandemic declaration and depression/anxiety among U.S. adults

**DOI:** 10.1371/journal.pone.0279963

**Published:** 2022-12-30

**Authors:** David Adzrago, Saanie Sulley, Ishmael Tagoe, Emmanuel Odame, Lohuwa Mamudu, Faustine Williams

**Affiliations:** 1 Center for Health Promotion and Prevention Research, The University of Texas School of Public Health, Houston, Texas, United States of America; 2 National Healthy Start Association, Washington, DC, United States of America; 3 School of Population Health, University of Toledo, Toledo, Ohio, United States of America; 4 Department of Environmental Health Sciences, School of Public Health, University of Alabama at Birmingham, Birmingham, Alabama, United States of America; 5 Department of Public Health, California State University, Fullerton, California, United States of America; 6 Division of Intramural Research, National Institute on Minority Health and Health Disparities, National Institutes of Health, Bethesda, Maryland, United States of America; University of Technology Sydney, AUSTRALIA

## Abstract

**Background:**

Although studies have investigated the impact of the COVID-19 on mental health, few studies have attempted to compare the prevalence of depression/anxiety symptoms among U.S. adults before and after the COVID-19 pandemic declaration. We examined the prevalence and association between depression/anxiety symptoms and COVID-19 pandemic declaration among U.S. adult population and subgroups.

**Methods:**

A nationally representative cross-sectional study of the Health Information National Trends Survey (HINTS 5, Cycle 4) assessing health-related information and behaviors in U.S. adults aged ≥18 years from February through June 2020. The primary dependent variable was **cur**rent depression/anxiety derived from Patient Health Questionnaire-4. The main independent variable was responses before and after the COVID-19 pandemic declaration in addition to sexual identity heterosexual identity, /race/ethnicity and rural-urban commuting areas. Covariates were sociodemographic factors, and health risk behaviors. Weighted percentages, multivariable logistic regression, and Chi-square tests were used to establish the prevalence and association between current depression/anxiety and the independent variables and covariates.

**Results:**

A total of 3,865 participants completed the survey and included 35.3% of the participants before the COVID-19 pandemic declaration. Most of the sample were aged 50–64 years [33.0%]; males [51.0%]; and non-Hispanic Whites [70.1%]). The post-pandemic declaration included participants, aged 35–49 years [27.0%]; females [52.6%]; and non-Hispanic Whites [59.6%]). The prevalence of depression/anxiety was higher after the COVID-19 pandemic declaration (32.2%) than before the declaration (29.9%). Higher risks of depression/anxiety symptoms after the declaration were associated with being a sexual minority ([adjusted odds ratio] AOR, 2.91 [95% confidence interval (CI), 1.38–6.14]) and having fair/poor general health (AOR, 2.91 [95% CI, 1.76–4.83]). The probability of experiencing depression/anxiety symptoms after the declaration was highest among homosexuals/lesbians/gays (65.6%) compared to bisexuals (39.6%), and heterosexuals (30.1%).

**Conclusions:**

In this study, young adults, non-Hispanic Whites, and those with fair/poor general health had a higher burden of depression/anxiety symptoms after the pandemic declaration. The development of psychological support strategies to promote wellbeing during the pandemic may reduce psychological distress in the population, especially among at-risk populations.

## Background

On March 11, 2020, the World Health Organization (WHO) declared the novel coronavirus SARS-CoV-2—the virus responsible for the COVID-19 outbreak a global pandemic [1]. At a news briefing, Dr. Tedros Adhanom Ghebreyesus, WHO Director-General, noted that over the past 2 weeks, the number of cases outside China increased 13-fold, and the number of countries reporting new cases have increased threefold. He emphasized that the WHO is “deeply concerned both by the alarming levels of spread and severity, and by the alarming levels of inaction.” He called on countries to take “urgent aggressive action” to protect their citizens to reduce transmission [1]. On March 13, 2020, by Proclamation 9994, the United States (U.S.) President declared a national emergency concerning the COVID-19 pandemic [2]. As a result, local, state, and federal governments implemented social distancing and social isolation, including stay-at-home, as well as shelter in place measures/orders to reduce the spread of the virus/disease. Despite the public health importance of these measures, evidence has shown that long-term isolation could have a profound effect on all aspects of society; including but not limited to economic hardships, as well as physical, psychosocial/mental health, and chronic diseases [3–7].

Studies on previous pandemics including Ebola, Middle East respiratory syndrome, severe respiratory syndrome, the influenza A as well as early published work on COVID-19 revealed a significant increase in mental health and distress [7, 8]. The sudden change in daily activities, social interactions, and the fear of being infected by the COVID-19 virus are major stressors that can increase the risk of mental health problems including depression/anxiety [9]. For instance, studies conducted among the general population, students, healthcare professionals, and other frontliners in Asia, Europe, and the Middle East indicated over 50% rise in depression, eating, and sleep disorders [10–22]. Although studies have investigated the impact of the COVID-19 on mental health, research that draws a comparison of the prevalence and association of depression/anxiety before and after the pandemic and emergency declaration in the U.S. is scarce.

Subsequently, our aims were (1) to examine the prevalence of depression/anxiety symptoms among the U.S. adults population before and after the COVID-19 pandemic declaration; (2) the association between depression/anxiety symptoms and COVID-19 pandemic declaration; and (3) whether the association between depression/anxiety and COVID-19 pandemic declaration varies between and within-population subgroups by sociodemographic characteristics and rural/urban residence type. The findings would provide an important understanding of the adverse effect of the COVID-19 pandemic declaration on mental health and help develop effective mental health support or outreach strategies to promote wellbeing during pandemics and reduce social risk and distress in the population.

## Methods

### Study population and data

We examined data from the 2020 Health Information National Trends Survey (HINTS 5, Cycle 4) de-identified publicly available file (https://hints.cancer.gov/data/download-data.aspx), a nationally representative annual cross-sectional survey of non-institutionalized adult population of the U.S., sponsored by the National Cancer Institute (NCI) since 2003 [23, 24]. The HINTS was designed to assess health-related information and health-related behaviors such as anxiety/depression symptoms and sociodemographic characteristics. The HINTS 5, Cycle 4 is the new publicly available data and included a sample of 3,865 adults, which were used for our study. The data was collected from February through June 2020. We wrote our paper using the Strengthening the Reporting of Observational Studies in Epidemiology (STROBE) reporting guideline [25].

### Measures

#### Dependent variable

The main dependent variable of interest was current depression/anxiety status and was derived from Patient Health Questionnaire-4 (PHQ-4) in the HINTS 5 survey. The PHQ-4 is a validated four-item on a four-point Likert-type scale, which assesses symptoms/signs of depression/anxiety [26, 27]. The total scores range from 0–12, where scores are rated as normal/negative (0–2), mild (3–5), moderate (6–8), and severe (9–12) [26, 27]. We dichotomized the current depression/anxiety status variable to indicate current depression/anxiety status as normal/no depression/anxiety and depression/anxiety (mild/moderate/severe).

#### Independent variables

The primary independent variable was responses before and after the WHO declaration of the COVID-19 pandemic. This variable was obtained from the HINTS 5, Cycle 4 methodology report (https://hints.cancer.gov/data/methodology-reports.aspx) [24]. From the report, this variable can be used to examine responses before and after COVID-19 became a concern in the U.S. [24]. It can also be used to assess the impact of COVID-19 [24]. Thus, this variable was determined based on the WHO declaration of COVID-19 as a pandemic on March 11, 2020. The HINTS 5, cycle 4 data was collected from February through June 2020. The HINTS 5, Cycle 4 survey responses that were received by Westat before the declaration, were categorized as responses before the COVID-19 pandemic declaration and responses after the WHO declaration of the COVID-19 pandemic declaration.

The secondary independent variables were self-report sexual identity (heterosexual/straight, homosexual/lesbian/gay, and bisexual), race/ethnicity (non-Hispanic White, non-Hispanic Black/African American, Hispanic, non-Hispanic Asian, and non-Hispanic other), and rural-urban commuting areas (metropolitan and micropolitan/small town/rural). In Models 2 and 3, we recoded sexual identity to two groups (i.e., heterosexual/straight and homosexual/lesbian/gay/bisexual) due to limited samples in this subgroup.

### Covariates

Based on previous studies [28–30], we analyzed the following covariates in our study. As part of the HINTS survey, participants provided self-reported information on age (18–25, 26–34, 35–49, 50–64, and ≥65), sex (male and female), level of education completed (<High School, high school graduate, some college, and college graduate or higher), total annual family income (<$20,000, $20,000-$34,999, $35,000-$49,999, $50,000-$74,999, and ≥$75,000), and employment status (employed or unemployed), health insurance (no or yes), ever been diagnosed with depression/anxiety disorder symptoms (no or yes), and cigarette smoking status (non-smoker, former smoker, and current smoker). For this analysis, the following variables were collapsed; marital status (single/never been married, married/living as married/living with a romantic partner, separated/divorced, and widowed), general health status (excellent/very good/good and fair/poor), number of days per week of moderate physical activity intensity (none and at least one day per week), binge drinking (never and at least one/more times), and body mass index (BMI) (underweight [<18.5], (healthy/normal weight [18.5–24.9], overweight [25.0–29.9], obese [≥30.0]).

### Ethical approval

No Institutional Review Board approval was required for this study because our analytic sample was derived from publicly available data through the NCI website at https://hints.cancer.gov/data/download-data.aspx.

### Statistical analyses

**Fig 1:**
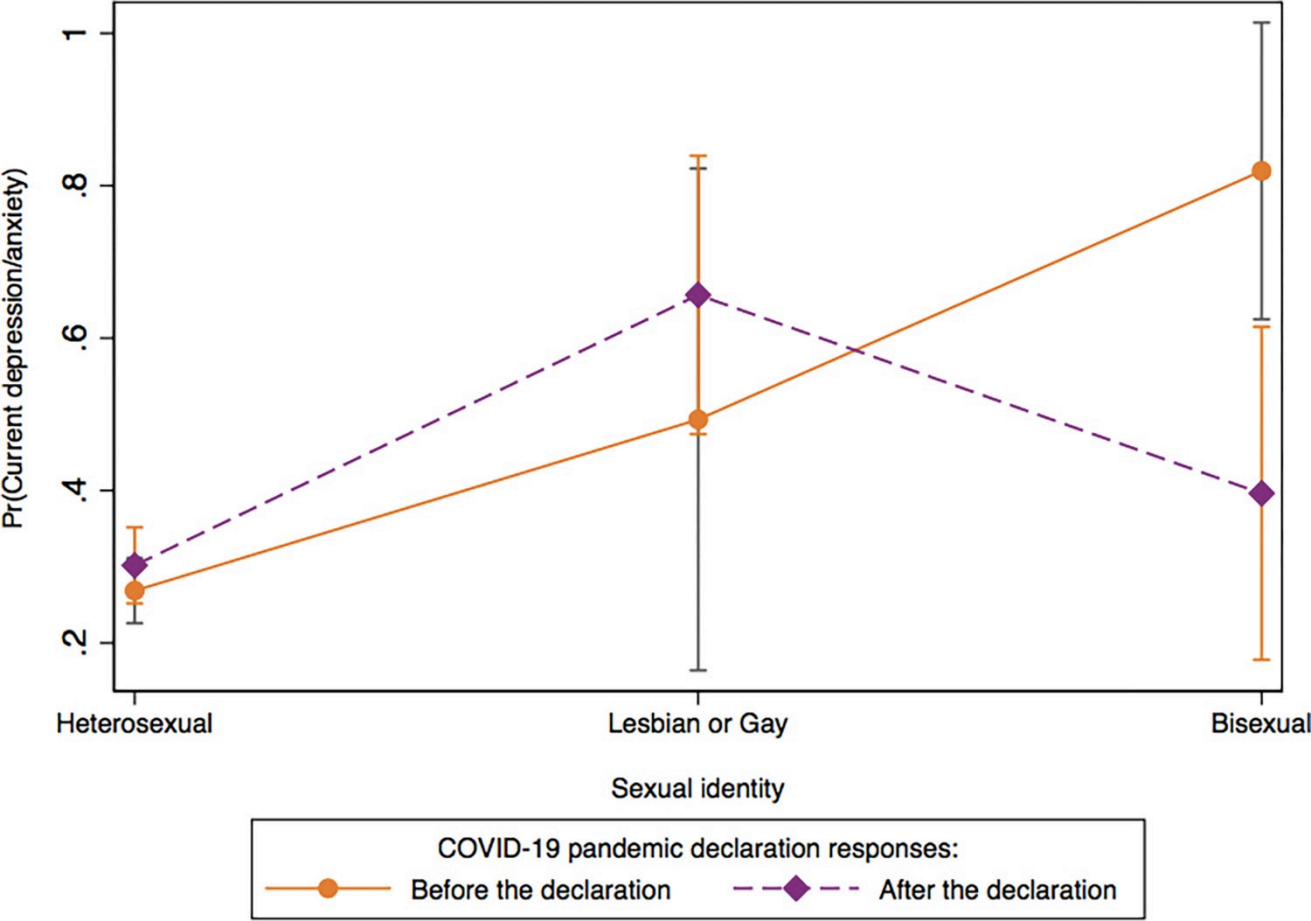
Differences in current depression/anxiety between and within sexual identity and COVID-19 pandemic declaration.

**Fig 2:**
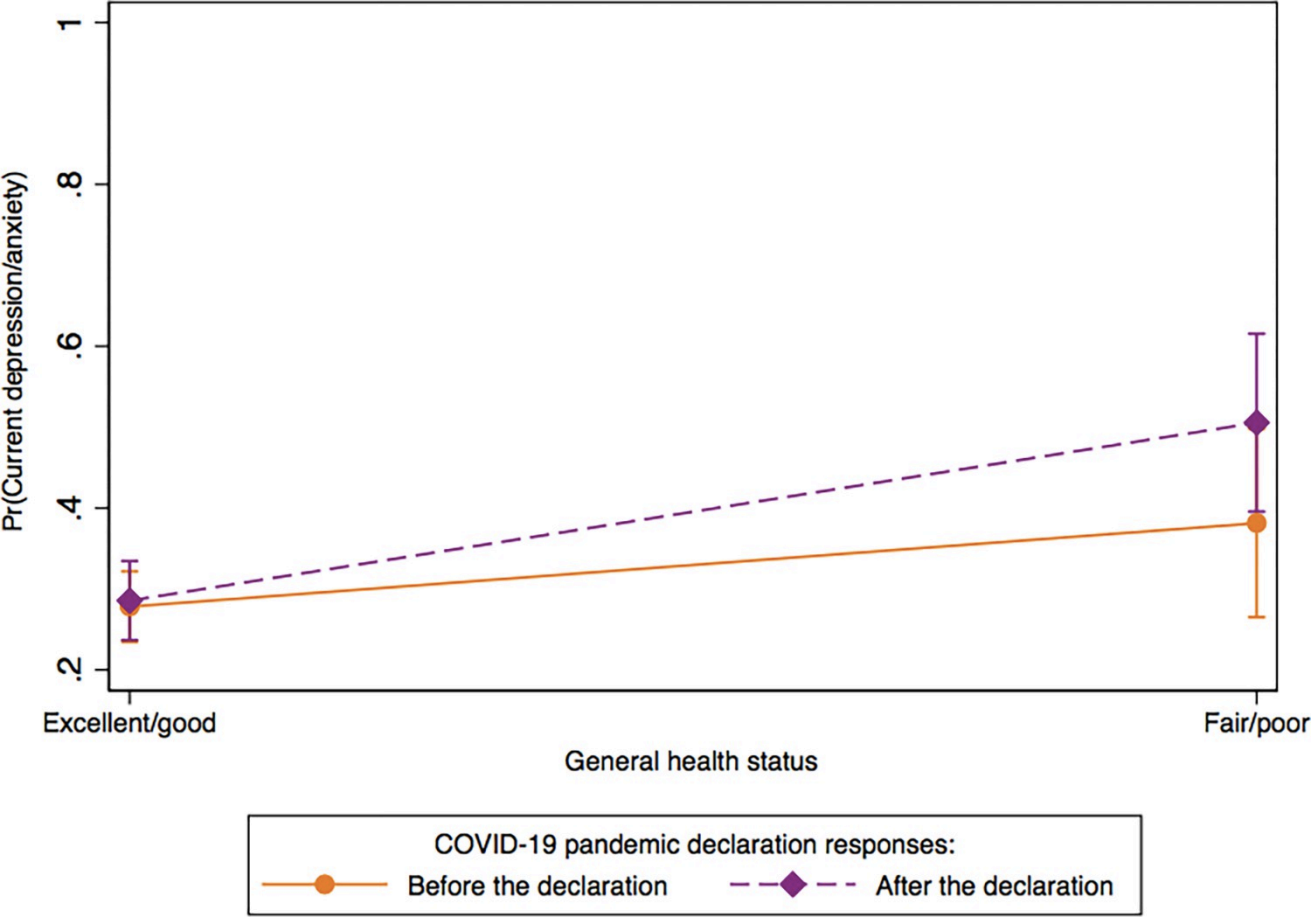
Differences in current depression/anxiety between and within general health status and COVID-19 pandemic declaration.

The data samples were weighted by sampling weight and replicate weight, according to recommendations described by the HINTS [24]. The weighted analyses offset non-response bias and produce nationally representative estimates [24]. Prevalence estimates of demographic and current depression/anxiety characteristics were examined to characterize the sample on responses before and after the COVID-19 pandemic declaration. In Model I, we used multivariable logistic regression to examine the association of current depression/anxiety in U.S. adults with COVID-19 pandemic declaration, sexual identity, race/ethnicity, and rural-urban commuting area, adjusting for the covariates (age, sex, level of education completed, total annual family income, and employment status, health insurance, depression/anxiety disorder symptoms, cigarette smoking status, marital status, general health status, moderate physical activity intensity, binge drinking, BMI). We further examined whether the association between depression/anxiety and COVID-19 pandemic declaration varies between and within-population subgroups (i.e. sexual identity, rural/urban commuting area, race/ethnicity, sex, employment, and general health). To perform between and within-group differences, we first examined the interactions between COVID-19 pandemic declaration and sexual identity, rural/urban commuting area, race/ethnicity, sex, employment, and general health, respectively, using joint significant effects with Adjusted Wald test. Next, we performed margin analyses to examine differences in current depression/anxiety between and within COVID-19 pandemic declaration and sexual identity (Fig 1), and differences in current depression/anxiety between and within COVID-19 pandemic declaration and general health status (Fig 2).

Additionally, in Model II and Model III we examined the independent variables and the covariates (i.e., age, sex, race/ethnicity, sexual identity, marital status, income, employment status, rural-urban commuting area, general health status, and physical activity) associated with depression/anxiety before and after the declaration of COVID-19 pandemic using multivariable logistic regression, respectively. All logistic regression models were based on complete cases analysis (i.e., listwise deletion). Results are reported using weighted percentages, adjusted odds ratios (AOR), 95% confidence intervals (CI) at 2-tailed, and statistically significant *p*-value at <0.05. We assessed multicollinearity among all the independent variables, there was no significant collinearity problems because our mean variance inflation factor was 1.19. Analyses were performed using STATA/SE, version 16.1 (StataCorp, 2019).

## Results

### Sample characteristics

Table 1 describes the sociodemographic, health behaviors, and depression/anxiety characteristics of the study sample by responses before and after the WHO declared COVID-19 a pandemic. A total of 64.6% of the sample provided responses after the COVID-19 pandemic declaration. Among the responses after the COVID-19 pandemic declaration, were aged 35–49 (27.0%), female (52.6%), non-Hispanic White (59.6%), married/living as married (52.4%), had some college degree (38.9%), a total family annual income of ≥$75,000 (40.8%), lived in the metropolitan commuting area (88.4%), and obese (35.4%). Slightly greater proportions of the sample after the COVID-19 pandemic declaration identified as lesbian/gay (2.9%) or bisexual (2.9%), had fair/poor general health (14.5%), unemployed (40.6%), no health insurance (9.4%), were not physically active (26.1%), current cigarette smoker (13.4%), engaged in binge drinking at least one time (43.7%), ever been diagnosed with depression/anxiety disorder (23.7%), and reported current mild/moderate/severe depression/anxiety symptoms (32.2%).

**Table 1:** Weighted descriptive statistics of U.S. adults stratified by response before and after the COVID-19 pandemic declaration, HINTS 5 Cycle 4.

Characteristics	Overall sample	Responses before the COVID-19 pandemic declaration	Responses after the COVID-19 pandemic declaration
	N (Weighted %, SE)	n (Weighted %, SE)	n (Weighted %, SE)
	3,865 (100%)	1,437 (35.3%, 0.01)	2,428 (64.6%, 0.01)
**Age**			
18–25	147 (13.2, 0.01)	41 (10.2, 0.02)	106 (14.9, 0.01)
26–34	337 (12.9, 0.01)	110 (9.6, 0.01)	227 (14.6, 0.01)
35–49	703 (25.5, 0.01)	212 (22.7, 0.02)	491 (27.0, 0.02)
50–64	1,142 (27.7, 0.01)	433 (33.0, 0.02)	709 (24.8, 0.01)
≥65	1,409 (20.5, <0.01)	598 (24.3, 0.01)	811 (18.4, 0.01)
**Sex**			
Female	2,204 (51.3, <0.01)	804 (49.00, 0.02)	1,400 (52.63, 0.01)
Male	1,561 (48.6, <0.01)	598 (51.00, 0.02)	963 (47.37, 0.01)
**Race/ethnicity**			
Non-Hispanic White	2,133 (63.3, <0.01)	904 (70.1, 0.02)	1,229 (59.6, 0.01)
Non-Hispanic Black	481 (11.1, <0.01)	135 (8.3, 0.01)	346 (12.6, 0.01)
Hispanic	596 (16.9, <0.01)	170 (12.8, 0.02)	426 (19.2, 0.01)
Non-Hispanic Asian	161 (5.2, <0.01)	51 (4.1, 0.01)	110 (5.8, 0.01)
Non-Hispanic other	119 (3.3, <0.01)	49 (4.5, 0.01)	70 (2.6, 0.01)
**Sexual identity**			
Heterosexual	3,402 (94.5, 0.01)	1,289 (95.3, 0.01)	2,113 (94.1, 0.01)
Homosexual/lesbian/gay	81 (2.6, 0.01)	26 (2.1, 0.01)	55 (2.9, 0.01)
Bisexual	82 (2.7, <0.01)	30 (2.4, 0.01)	52 (2.9, 0.01)
**Marital status**			
Single/never married	645 (30.7, <0.01)	212 (25.4, 0.02)	433 (33.7, 0.01)
Married/living as married	1,978 (54.7, <0.01)	750 (59.0, 0.02)	1,228 (52.4, 0.01)
Divorced/separated	684 (9.7, <0.01)	265 (10.2, 0.01)	419 (9.5, 0.01)
Widowed	414 (4.6, <0.01)	166 (5.2, 0.01)	248 (4.2, <0.01)
**Level of education completed**			
Less than High School	273 (8.0, 0.01)	90 (7.20, 0.01)	183 (8.4, 0.01)
High School graduate	705 (22.5, 0.01)	251 (20.2, 0.02)	454 (23.7, 0.01)
Some college	1,081 (39.1, 0.01)	415 (39.6, 0.03)	666 (38.9, 0.01)
College graduate/higher	1,663 (30.2, <0.01)	643 (32.9, 0.02)	1,020 (28.8, 0.01)
**Total annual family income**			
<$20,000	624 (15.1, 0.01)	216 (14.1, 0.01)	408 (15.6, 0.01)
$20,000 - $34,999	451 (11.4, 0.01)	170 (10.8, 0.02)	281 (11.8, 0.01)
$35,000 - $49,999	460 (12.6, 0.01)	166 (12.0, 0.02)	294 (13.0, 0.01)
$50,000 - $74,999	592 (18.2, 0.01)	229 (17.4, 0.02)	363 (1.7, 0.02)
≥$75,000	1,321 (42.4, 0.02)	514 (45.5, 0.03)	807 (4.8, 0.02)
**General health status**			
Excellent/very good/good	3,192 (85.8, 0.01)	1,200 (86.7, 0.02)	1,992 (85.4, 0.01)
Fair/poor	627 (14.1, 0.01)	221 (13.3, 0.02)	406 (14.5, 0.01)
**Employment status**			
Unemployed	1,888 (40.8, 0.01)	756 (41.3, 0.02)	1,132 (40.6, 0.02)
Employed	1,890 (59.1, 0.01)	652 (58.7, 0.02)	1,238 (59.3, 0.02)
**Health insurance**			
No	203 (9.0, <0.01)	63 (8.2, 0.01)	140 (9.4, 0.01)
Yes	3,604 (91.0, <0.01)	1,352 (91.7, 0.01)	2,252 (90.5, 0.01)
**Rural-urban commuting area**			
Metropolitan	3,387 (87.1, 0.01)	1,217 (84.8, 0.01)	2,170 (88.4, 0.01)
Micropolitan/small town/rural	478 (12.8, 0.01)	220 (15.1, 0.01)	258 (11.5, 0.01)
**Body mass index (BMI)**			
Normal weight (18.5–24.9)	1,143 (32.8, 0.01)	438 (32.3, 0.03)	705 (33.1, 0.02)
Underweight (<18.5)	63 (1.2, <0.01)	22 (1.3, <0.01)	41 (1.1, <0.01)
Overweight (25.0–29.9)	1,265 (31.7, 0.01)	492 (34.4, 0.02)	773 (30.2, 0.02)
Obese (≥30.0)	1,274 (34.1, 0.01)	454 (31.8, 0.03)	820 (35.4, 0.02)
**Moderate physical activity intensity**			
None	1,048 (32.8, 0.01)	392 (32.3, 0.02)	656 (33.1, 0.01)
At least one day per week	2,750 (1.24, 0.01)	1,021 (1.3, 0.02)	1,729 (1.18, 0.01)
**Cigarette smoking status**			
Never	2,422 (63.1, 0.01)	884 (60.5, 0.02)	1,538 (64.5, 0.02)
Former smoker	935 (23.0, 0.01)	372 (24.9, 0.02)	563 (22.0, 0.02)
Current smoker	436 (13.8, 0.01)	158 (14.5, 0.02)	278 (13.4, 0.01)
**Binge drinking**			
Never	3,192 (79.6, 0.01)	1,190 (78.6, 0.02)	2,002 (80.2, 0.01)
One/more times	673 (20.3, 0.01)	247 (21.3, 0.02)	426 (19.7, 0.01)
**Ever been diagnosed with depression/anxiety disorder**			
No	2,897 (75.6, 0.01)	1,086 (74.58, 0.02)	1,811 (76.2, 0.01)
Yes	908 (24.3, 0.01)	338 (25.42, 0.02)	570 (23.7, 0.01)
**Current depression/anxiety status**			
None	2,670 (68.5, 0.01)	1,003 (70.0, 0.02)	1,667 (67.7, 0.02)
Mild/moderate/severe	1,060 (31.4, 0.01)	387 (29.9, 0.02)	673 (32.2, 0.02)

Data Source: 2020 Health Information National Trends Survey (HINTS 5, Cycle 4.

Unweighted N = 3,865, and Weighted N = 253815197

SE = Standard error.

### Multivariable logistic regression

The multivariable logistic regression examining the association of current depression/anxiety with sociodemographic factors is shown in Table 2. Individuals aged ≥65 years (AOR, 0.33 [95% CI, 0.14–0.77]). were less likely to experience current depression/anxiety symptoms compared with those aged 18–25 years. Non-Hispanic Blacks (AOR, 0.59 [95% CI, 0.39–0.89]) and non-Hispanic Asians (AOR, 0.44 [95% CI, 0.21–0.89]) were less likely to experience depression/anxiety symptoms compared to non-Hispanic Whites. Homosexual/lesbian/gay (AOR, 4.45 [95% CI, 2.06–9.59]) and bisexual (AOR, 2.90 [95% CI, 1.09–7.72]) were more likely to experience depression/anxiety symptoms compared to heterosexual individuals. The population with a total annual family income of ≥$75,000 (AOR, 0.59 [95% CI, 0.35–0.99]) was less likely to experience depression/anxiety symptoms compared to those with a total annual family income of <$20,000. Unemployed individuals (AOR, 1.58 [95% CI, 1.01–2.47]) were more likely to experience depression/anxiety symptoms compared to employed individuals. Those who self-rated their general health as fair/poor (AOR, 2.39 [95% CI, 1.55–3.67]) were more likely to experience depression/anxiety symptoms compared to those who rated theirs as excellent/very good/good. Additionally, those who reported being physically active (AOR, 0.63 [95% CI, 0.44–0.90]) were less likely to experience depression/anxiety symptoms compared to those who did not engage in any moderate physical activity intensity. Contrary to our hypothesis, we did not find any significant overall differences in depression/anxiety symptoms between responses before and after the COVID-19 pandemic declaration.

**Table 2:** Association of current depression/anxiety with sociodemographic, health characteristics and COVID-19 pandemic declaration among U.S. adults aged ≥18 years, HINTS 5 Cycle 4 -Model I.

	AOR	95% CI
**Age**		
18–25	Ref	-
26–34	1.26	(0.64–2.49)
35–49	1.02	(0.53–1.99)
50–64	0.70	(0.35–1.41)
≥65	**0.33** **	**(0.14–0.77)**
Sex		
Female	Ref	-
Male	0.78	(0.56–1.08)
**Race/ethnicity**		
Non-Hispanic White	Ref	-
Non-Hispanic Black	**0.59** **	(0.39–0.89)
Hispanic	0.93	(0.62–1.39)
Non-Hispanic Asian	**0.44** *	(0.21–0.89)
Non-Hispanic other	1.83	(0.81–4.10)
**Sexual identity**		
Heterosexual	Ref	-
Homosexual/lesbian/gay	**4.45** ***	(2.06–9.59)
Bisexual	**2.90** *	(1.09–7.72)
**Marital status**		
Single/never married	Ref	-
Married/living as married	0.86	(0.55–1.36)
Divorced/separated	1.24	(0.70–2.20)
Widowed	1.43	(0.71–2.91)
**Total family annual income**		
<$20,000	Ref	-
$20,000 - $34,999	0.59	(0.34–1.05)
$35,000 - $49,999	0.60	(0.31–1.15)
$50,000 - $74,999	0.68	(0.38–1.20)
≥$75,000	**0.59** *	**(0.35–0.99)**
**Employment status**		
Employed	Ref	-
Unemployed	**1.58** *	**(1.01–2.47)**
**Rural-urban commuting area**		
Metropolitan	Ref	-
Micropolitan/Small town/rural	0.87	(0.60–1.27)
**General health status**		
Excellent/very good/good	Ref	-
Fair/poor	**2.39** ***	**(1.55–3.67)**
**Moderate physical activity intensity**		
None	Ref	-
At least one day per week	**0.63** **	**(0.44–0.90)**
**COVID-19 declaration**		
Responses before the declaration	Ref	-
Responses after the declaration	1.13	(0.80–1.60)

Data Source: 2020 Health Information National Trends Survey, (HINTS 5, Cycle 4).

Unweighted N = 3,865, and Weighted N = 253815197

AOR = Adjusted odds ratio.

95% CI = 95% confidence interval.

Ref = Reference group.

*p ≤0.05

**p ≤0.01

***p ≤ 0.001.

Table 3 shows depression/anxiety symptoms before and after the COVID-19 pandemic declaration, respectively. Compared with heterosexual individuals, homosexual/lesbian/gay/bisexual individuals had higher odds of depression/anxiety symptoms before the COVID-19 pandemic declaration (AOR, 5.76 [95% CI, 2.21–15.00]) and after the pandemic declaration (AOR, 2.91 [95% CI, 1.38–6.14]). Engaging in moderate physical activity intensity of at least one day per week was associated with lower odds of depression/anxiety symptoms compared with not engaging in moderate physical activity intensity before the declaration (AOR, 0.54 [95% CI, 0.34–0.87]) and after the declaration (AOR, 0.66 [95% CI, 0.44–0.99]). Compared with individuals with total family annual income of <$20,000, individuals who reported family annual income of $20,000-$34,999 (AOR, 0.41 [95% CI, 0.18–0.97]) and ≥$75,000 (AOR, 0.45 [95% CI, 0.21–0.96) had lower odds of reporting depression/anxiety symptoms. Similarly, compared to those aged 18–25 years, the older population (≥65 years) had decreased risk of having depression/anxiety symptoms after the pandemic declaration (AOR, 0.30 [95% CI, 0.12–0.74]). Non-Hispanic Black (AOR, 0.58 [95% CI, 0.36–0.95]) and non-Hispanic Asian individuals (AOR, 0.40 [95% CI, 0.18–0.91]) had lower odds of depression/anxiety symptoms compared with non-Hispanic White individuals after the declaration. As expected, individuals with fair/poor general health had increased odds (AOR, 2.91 [95% CI, 1.76–4.83]) of having depression/anxiety symptoms after the pandemic declaration when compared to those who reported excellent/very good/good general health.

**Table 3:** Odd ratios and confidence intervals for associations between current depression/anxiety with sociodemographic and health characteristics stratified by before and after the declaration of COVID-19 pandemic among U.S. adults aged ≥18 years, HINTS 5 Cycle 4.

	Before the COVID-19 pandemic declaration (Model II)		After the COVID-19 pandemic declaration (Model III)	
	AOR	95% CI	AOR	95% CI
**Age**				
18–25	Ref	-		Ref
26–34	1.86	(0.60–5.74)	1.10	(0.56–2.18)
35–49	1.33	(0.41–4.35)	0.96	(0.48–1.92)
50–64	1.05	(0.33–3.38)	0.59	(0.28–1.24)
≥65	0.43	(0.16–1.20)	**0.30** ***	**(0.12–0.74)**
**Sex**				
Female	Ref	-		Ref
Male	0.82	(0.52–1.28)	0.80	(0.54–1.19)
**Race/ethnicity**				
Non-Hispanic White	Ref	-		Ref
Non-Hispanic Black	0.59	(0.24–1.45)	**0.58** *	**(0.36–0.95)**
Hispanic	1.48	(0.74–2.97)	0.77	(0.46–1.27)
Non-Hispanic Asian	0.47	(0.15–1.47)	**0.40** *	**(0.18–0.91)**
Non-Hispanic other	3.61	(0.95–13.76)	1.08	(0.47–2.50)
**Sexual identity**				
Heterosexual	Ref	-		Ref
Homosexual/lesbian/gay	**5.76** ***	**(2.21–15.00)**	**2.91** ***	**(1.38–6.14)**
Bisexual				
**Marital status**	Ref	-		Ref
Single/never married	0.77	(0.34–1.78)	0.92	(0.58–1.47)
Married/living as married	1.53	(0.66–3.55)	1.09	(0.58–2.05)
Divorced/separated	1.50	(0.50–4.51)	1.37	(0.64–2.91)
Widowed				
**Total family annual income**	Ref			Ref
<$20,000	0.41*	**(0.18–0.97)**	0.67	(0.33–1.34)
$20,000 - $34,999	0.61	(0.28–1.36)	0.55	(0.26–1.17)
$35,000 - $49,999	0.61	(0.28–1.33)	0.68	(0.35–1.33)
$50,000 - $74,999	0.45*	**(0.21–0.96)**	0.61	(0.31–1.22)
≥$75,000				
**Employment status**	Ref			Ref
Employed	1.61	(0.96–2.71)	1.54	(0.94–2.54)
Unemployed				
**Rural-urban commuting area**	Ref			Ref
Metropolitan	0.78	(0.41–1.49)	0.95	(0.60–1.51)
Micropolitan/Small town/rural				
**General health status**	Ref			Ref
Excellent/very good/good	1.46	(0.86–2.48)	**2.91** ***	**(1.76–4.83)**
Fair/poor				
**Moderate physical activity intensity**	Ref			Ref
None	**0.54** **	**(0.34–0.87)**	**0.66** *	**(0.44–0.99)**
At least one day per week	Ref	-		Ref

Data Source: 2020 Health Information National Trends Survey (HINTS 5, Cycle 4).

Unweighted N = 3,865, and Weighted N = 253815197

**Model II** (if before the declaration of COVID-19 pandemic) = independent variables + covariates.

**Model III** (if after the declaration of COVID-19 pandemic) = independent variables + covariates.

AOR = Adjusted odds ratio. 95% CI = 95% confidence interval.

Ref = Reference group.

*p ≤0.05

**p ≤0.01

***p ≤0.001

### Between and within current depression/anxiety comparison

Fig 1 describes the differences in current depression/anxiety between and within sexual identity and the pandemic declaration. Results revealed that between the sexual identity subgroups, individuals who self-identified as bisexuals (81.9%) had the highest probability of experiencing depression/anxiety symptoms before the pandemic declaration compared to lesbians/gays (49.3%) and heterosexuals (26.8%). Examining responses after the pandemic declaration showed a 16.3% and 3.3% increase in current depression/anxiety symptoms among lesbians/gays (49.3 vs. 65.6) and heterosexuals (26.8 vs. 30.1%) respectively. Whereas the probability of depression/anxiety symptoms among bisexuals (39.6%) was higher compared to heterosexuals, it was lower than before the pandemic declaration.

Fig 2 depicts that the probability of experiencing depression/anxiety symptoms was higher after the declaration than before. Before the pandemic declaration, individuals who rated their general health as fair/poor had a significantly higher probability of experiencing depression/anxiety symptoms compared to those who rated their health as excellent/good (38.1% vs. 27.8%). After the pandemic declaration, the probability of experiencing depression/anxiety symptoms significantly increased among those who rated their general health as fair/poor compared to those who rated theirs as excellent/good (50.5% vs. 28.5%). We found significant interaction only between COVID-19 pandemic declaration and sexual identity (*p* = 0.002) and general health (*p* = 0.001).

## Discussion

To our knowledge, this is the first study to examine the association between depression/anxiety and stay-at-home orders following the WHO and the national emergency declaration by the U.S. President on March 11^th^ and 13^th^ 2020, while accounting for sociodemographic characteristics and place of residence. We found that being a sexual minority (i.e., homosexual/lesbian/gay/bisexual) was strongly associated with depression/anxiety symptoms. For instance, lesbians/gays/bisexuals were 191% more likely to report depression/anxiety symptoms, relative to heterosexuals. Total family income, employment status, general health, and physical activity elevated depression/anxiety symptom levels among the U.S. adult population after the WHO and the national emergency pandemic declaration in March 2020.

According to the National Center for Health Statistics, the prevalence of depression/anxiety was higher during the pandemic than before (i.e., 6.5% and 8.1%, respectively) [31]. Ettman et al. [12] used the AmeriSpeak panel and the National Health and Nutrition Examination Survey (NHANES) to assess the prevalence and factors associated with depression symptoms among U.S. adults during and before the COVID-19 pandemic. The authors reported that the severity of depression symptoms was significantly higher during the pandemic than before. A systematic review [32] on the effect of the pandemic on psychiatric disorders and suicide rates revealed a significant association between the COVID-19 pandemic and high levels of distress, anxiety, fear of contagion, depression, and abnormal sleep in the general population and among healthcare workers. Consistent with these findings, we found that approximately a third (i.e., 31.4%) of the study population reported current mild/moderate/severe depression/anxiety symptoms during the pandemic. Additionally, we found a 2.2% increase in the proportion of U.S. adults who reported experiencing depression/anxiety after the pandemic declaration (i.e., 32.2% vs. 29.9%). Even though the multivariate logistic regression did not reveal any significant association between COVID-19 pandemic declaration and depression/anxiety symptoms in the overall U.S. adult population, significant differences exist in some sub-populations. For instance, young adults, non-Hispanic Whites, those with poor/fair health were more likely to report depression/anxiety symptoms while the elderly, people who engaged in moderate physical activity, non-Hispanic Blacks, and Asians were less likely to report depression/anxiety symptoms. Moreover, self-reported depression/anxiety symptoms remained significantly higher among sexual minorities than in heterosexuals, although the odds were higher before the pandemic declaration.

Sociodemographic factors including age, race/ethnicity, and sexual identity have been reported to affect depression/anxiety during the pandemic [11–13, 16, 21, 33–40]. Czeisler et al. [34] [N = 5,573] analyzed cross-sectional nationally representative data on adults aged ≥18 years in Australia and U.S. regarding public support for stay-at-home orders and coping strategies. Results indicated a higher prevalence of depression/anxiety among younger individuals and women staying at home or in quarantine relative to those not in quarantine. Lofrano-Prado et al. [35] investigated the impact of stay-at-home orders and social distancing on mental health and found that women experienced increased anxiety, depression, low self-esteem, sadness, and stress compared to men. Similar results were reported in other countries, including Australia [16], India [39], Italy [15], Philippines [37], Spain [13, 17], and Turkey [21]. Our study revealed that U.S. adults aged ≥65 years were 70% less likely to report current depression/anxiety when compared to younger adults aged 18–25 years. We observed no statistically significant association between COVID-19 declaration and sex, nor was there an association found with marital status. The present study further showed that both non-Hispanic Black and non-Hispanic Asian had decreased odds of experiencing current depression/anxiety symptoms (42% vs. 60%, respectively). Earlier studies suggested depression/anxiety among homosexuals/lesbian/gay and bisexual individuals was higher than among heterosexual individuals [41, 42]. Consistent with published studies, those self-identified as homosexual/lesbian/gay and bisexual had the highest probability for depression/anxiety symptoms despite a slight increase in heterosexual responses after the declaration.

The burden of the COVID-19 pandemic has resulted in the loss of work, income, and disruption of social life, and associated high psychological distress worldwide. Regarding socio-economic factors, our study found that individuals with a total family annual income of ≥$75,000 had a 41% decreased odds of experiencing current depression/anxiety while unemployed individuals had a 58% increased odds of current depression. These findings are consistent with other studies that showed an increase in anxiety/depression because of restrictions placed on activities during the pandemic [38, 43].

COVID-19 pandemic social distancing and stay-at-home orders did not only overwhelm the delivery of healthcare around the world but some individuals with underlying conditions were compelled to delay/avoid seeking healthcare due to the lockdown. Unsurprisingly, our analysis indicated that individuals with fair/poor health had 191% higher odds of current depression/anxiety relative to before the declaration. As noted in previous research, regular physical activity improves immune system function, and it is likely to provide protective buffers and reduce stress, as well as prevents the onset of chronic diseases and mental health conditions [44, 45]. Correspondingly, those who reported engaging in moderate physical activity at least once per week were 34% less likely to experience current depression/anxiety after the pandemic declaration. These findings are important for understanding the adverse effect of the COVID-19 pandemic declaration on the mental health of U.S. adults, especially among homosexuals/lesbians/gay/bisexuals and those in poor health.

### Limitations

This study has some limitations. First, the HINTS is a cross-sectional survey, and therefore, we were unable to establish any causal relationship between the WHO COVID-19 pandemic declaration and anxiety/depression. Second, HINTS did not include questions that specifically assess COVID-19 outcomes among the participants. Furthermore, the data is self-reported and may result in recall and social desirability biases. Also, the HINTS 5, cycle 4 data was collected from February through June 2020, hence, we were able to account for changes in the prevalence of depression/anxiety after June 2020, which may explain the temporal sequence of depression/anxiety across time.

## Conclusions

In this population-based, nationally representative sample of U.S. adults, we found that the COVID-19 declaration had a varied impact on the population. Overall, young adults, non-Hispanic Blacks, non-Hispanic Asian, and individuals with fair/poor self-reported health status, had higher odds of depression/anxiety symptoms after the pandemic declaration. Further, the high risk of depression/anxiety among sexual minority groups highlights the need to develop sustainable, community-focused, and community-led efforts to alleviate some of our society’s mental health problems. Moreover, the development of psychological support strategies to promote wellbeing during and after the pandemic may reduce psychological distress in the population, especially among at-risk populations.
